# Perineuronal net digestion with chondroitinase restores memory in mice with tau pathology

**DOI:** 10.1016/j.expneurol.2014.11.013

**Published:** 2015-03

**Authors:** Sujeong Yang, Matthias Cacquevel, Lisa M. Saksida, Timothy J. Bussey, Bernard L. Schneider, Patrick Aebischer, Riccardo Melani, Tommaso Pizzorusso, James W. Fawcett, Maria Grazia Spillantini

**Affiliations:** a John Van Geest Centre for Brain Repair, University of Cambridge, Robinson Way, Cambridge CB2 0PY, United Kingdom; b Brain Mind Institute, Ecole Polytechnique Fédérale de Lausanne, Lausanne 1015, Switzerland; c Department of Experimental Psychology, University of Cambridge, Downing Street, Cambridge CB2 3EB, United Kingdom; d MRC and Wellcome Trust Behavioural and Clinical Neuroscience Institute, University of Cambridge, Downing Street, Cambridge CB2 3EB, United Kingdom; e Inst Neuroscience CNR, via Moruzzi 1, 56125 Pisa, Italy; f NEUROFARBA Dept, University of Florence, Area S. Salvi Pad. 26, 50135 Florence, Italy

**Keywords:** Chondroitinase ABC, Microtubule-associated protein, Tauopathy, Memory dementia perineuronal net, Object recognition TEST

## Abstract

Alzheimer's disease is the most prevalent tauopathy and cause of dementia. We investigate the hypothesis that reactivation of plasticity can restore function in the presence of neuronal damage resulting from tauopathy. We investigated two models with tau hyperphosphorylation, aggregation and neurodegeneration: a transgenic mouse model in which the mutant P301S tau is expressed in neurons (Tg P301S), and a model in which an adeno-associated virus expressing P301S tau (AAV-P301S) was injected in the perirhinal cortex, a region critical for object recognition (OR) memory. Both models show profound loss of OR memory despite only 15% neuronal loss in the Tg P301S and 26% in AAV-P301S-injected mice. Recordings from perirhinal cortex slices of 3 month-old P301S transgenic mice showed a diminution in synaptic transmission following temporal stimulation. Chondroitinase ABC (ChABC) can reactivate plasticity and affect memory through actions on perineuronal nets. ChABC was injected into the perirhinal cortex and animals were tested for OR memory 1 week later, demonstrating restoration of OR memory to normal levels. Synaptic transmission indicated by fEPSP amplitude was restored to control levels following ChABC treatment. ChABC did not affect the progression of neurodegenerative tauopathy. These findings suggest that increasing plasticity by manipulation of perineuronal nets offers a novel therapeutic approach to the treatment of memory loss in neurodegenerative disorders.

## Highlights

• Progressive tau pathology in the perirhinal cortex impairs object recognition memory.
• ChABC treatment improves plasticity and object recognition memory in mice with tau pathology.
• The improvement of object recognition memory by ChABC is temporary and related to ChABC expression.

## Introduction

Perineuronal nets (PNNs) are extracellular matrix structures found predominantly around inhibitory parvalbumin-positive interneurons in the cortex and other brain regions. They play a part in the termination of developmental critical periods, and their degradation restores plasticity and enhances recovery from various types of CNS damage (Frischknecht and Gundelfinger, 2012; Kwok et al., 2011; Wlodarczyk et al., 2011). While PNN digestion with chondroitinase ABC (ChABC) has enabled restoration of function after focal lesions such as stroke and spinal cord injury (Bradbury et al., 2002; Soleman et al., 2012), it is not established whether re-activation of plasticity by ChABC might restore CNS function following the diffuse neuronal loss and damage that accompanies neurodegenerative disease caused by tau pathology. Loss of memory is a feature of many neurodegenerative diseases, and PNNs have been implicated in memory mechanisms (Gogolla et al., 2009; Romberg et al., 2013). We recently showed that digestion of PNNs in the perirhinal cortex (PRh), a structure critical for object recognition (OR) memory (Bartko et al., 2007; Mumby and Pinel, 1994; Winters et al., 2004), greatly prolonged long-term OR memory in wild type (WT) mice, and an identical memory prolongation was seen in mice lacking CNS Crtl1/Hapln1, an essential component of PNNs (Romberg et al., 2013). As the PRh is affected and recognition memory is impaired in human tauopathies such as Alzheimer's and Pick's diseases (Braak and Braak, 1991, 1992; Gold and Budson, 2008; Hornberger and Piguet, 2012), these results suggest that manipulation of PNNs could be a possible method for restoration of memory and compensation for neuronal dysfunction and loss in such diseases. In the present study, we tested whether PNN digestion can restore memory in the presence of the diffuse lesion produced by tau pathology. We used two lesion models: 1. transgenic mice expressing human tau with the P301S mutation, responsible for cases of frontotemporal dementia and parkinsonism linked to chromosome 17 (FTDP-17) (Spillantini and Goedert, 2013). These transgenic mice show progressive tau pathology in both central and peripheral nervous systems (Allen et al., 2002; Delobel et al., 2008; Mellone et al., 2013). 2. Mice in which an AAV expressing the human P301S tau was injected into the PRh of adult C57BL/6S mice. The localized tauopathy in this model avoids motor confounds, which occur with damage to motoneuons in transgenic P301S tau mice after six months.

Both Tg P301S mice and AAV P301S-injected mice developed a progressive tauopathy and a severe deficit in OR memory together with a deficit in synaptic transmission. Digestion of PNNs in the PRh with chondroitinase ABC (ChABC) – which targets the chondroitin sulphate proteoglycans (CSPGs), a major component of PNNs, critically responsible for their effect on plasticity (Carulli et al., 2010; Dityatev et al., 2010; Frischknecht et al., 2009; Kwok et al., 2012) – fully restored OR memory and synaptic transmission. These findings indicate that modification of PNNs can enable transient functional recovery from CNS dysfunction caused by diffuse neurodegeneration.

## Materials and methods

### Mice

Homozygous transgenic male mice expressing human mutant P301S tau (Tg P301S) under the control of the murine *Thy1* promoter were used for this study. In humans, the presence of the P301S mutation in the Tau gene leads to a progressive tauopathy causing frontotemporal dementia (Bugiani et al., 1999). This mouse model presents progressive tau pathology with the first tau deposits in homozygous mice appearing at 2 months of age and developing until the age of 5–6 months when the mice have to be culled due to their motor phenotype (Allen et al., 2002; Delobel et al., 2008). Age- and sex-matched C57BL/6S mice (Harlan, UK) were used as controls. Behavioral studies were performed in male mice at 1, 2 and 3 months of age, before the development of the overt motor phenotype (number of animals: 1 month: WT *n* = 7, P301S *n* = 9; 2 months: WT *n* = 5, P301S *n* = 7; 3 months: WT, *n* = 6 P301S *n* = 10). In order to ensure the specificity of the transgene effect in the behavioral deficit, heterozygous male Tg P301S mice (P301S Het) which start to develop tau pathology at the age of 7 months and have to be culled at the age of 14–15 months, were tested with their littermate controls at 3 and 7 months of age (number of animals: 3 months: WT *n* = 6, P301S Het *n* = 7; 7 months: WT *n* = 8, P301S Het *n* = 7). For AAV injection 8–9 week old male C57BL/6S mice (Harlan, UK) were used. Animals had unrestricted access to food and water, and were maintained on a 12 h light/dark cycle (lights off at 7:00 P.M.). All behavioral testing was conducted during the light phase of the cycle. All experiments were carried out in accordance with the UK Home Office Regulations for the Care and Use of Laboratory Animals and the UK Animals (Scientific Procedures) Act 1986.

### Spontaneous object recognition task

The spontaneous object recognition (OR) task was conducted as previously described for mice (Romberg et al., 2013), using a Y-shaped apparatus adapted for mice. The Y-apparatus had high, homogenous white walls constructed from Perspex to prevent the mouse from looking out into the room, thereby maximizing attention to the stimuli. One arm was used as the start arm, and the other two arms were used to display the objects (dimensions approximately 10 cm × 4 cm × 4 cm). All walls were 30 cm high, and each arm was 15 cm in length and 8 cm wide. A lamp illuminated the apparatus and a video camera was mounted 50 cm above the apparatus to record trials for analysis. All mice were habituated in three consecutive daily sessions in which they were placed in the start arm and left to explore the empty Y-apparatus for 5 min. The following test sessions were separated by a minimum of 48 h. Each test session consisted of a sample phase and a choice phase. In the sample phase, the animal was placed in the start arm and left to explore the two identical objects, which were placed on the end of two arms for 5 min. The choice phase followed after a delay of either 1 min or 3 h which the animal spent in the home cage. The choice phase was procedurally identical to the sample phase, except that one arm contained a novel object, whereas the other arm contained a copy of the object which was presented in the sample phase. An unused copy of the repeated object was used in order to avoid olfactory disturbance. Each animal received 2 test sessions for each delay. A different object pair was used for each session for a given animal, and the order of exposure to object pairs as well as the designated sample and novel objects for each pair were counterbalanced within and across groups. This test was performed 7 days after ChABC injections. The object exploration time was assessed from video recordings of the sample and choice phase. The direct nasal or head contacts only were regarded as an exploratory behavior. For the choice phase, a discrimination ratio was calculated by dividing the difference in exploration of the novel and familiar objects by the total object exploration time. Therefore, the discrimination ratio varies from 0 (equal exploration for novel and familiar objects) to 1 (exploration of the novel object only). The mean discrimination ratio across two test sessions was calculated for each animal. Group means were compared by ANOVA followed by Tukey post-hoc test with a significance level of *p* < 0.05, using GraphPad Prism version 5.0. A two-tailed *t*-test was used for two-group comparisons. (Number of mice for P301S homozygous study: P-nase *n* = 16, ChABC *n* = 13; Control: P-nase *n* = 7, ChABC *n* = 5; number of mice for P301S heterozygous study: P-nase *n* = 4, ChABC *n* = 4; Control: P-nase *n* = 4, ChABC *n* = 3; number of mice for the AAV-fpmax study: P-nase *n* = 6, ChABC *n* = 6; AAV-TauWT: P-nase *n* = 6, ChABC *n* = 6; AAV-P301S: P-nase *n* = 6, ChABC *n* = 6).

### ChABC injections

Protease-free ChABC (Seikagaku, Japan) or penicillinase (P-nase; Sigma-Aldrich, UK) as a non-relevant control enzyme was dissolved in 0.1% BSA to 50 U/ml of concentration and filtered through a 0.2 micron filter. Six injections in the PRh (3 per hemisphere, 0.5 μl with a speed of 0.2 μl/min) were performed stereotaxically under isoflurane anesthesia with a 10 μl Hamilton syringe and a 33 gauge needle at the following sites (in mm from bregma and the surface of skull): 1. anterior–posterior (AP): − 1.8; lateral (L): ± 4.6; ventral (V): − 4.4, 2. AP: − 2.8; L: ± 4.8; V: − 4.3 and 3. AP: − 3.8; L: ± 4.8; V: − 3.8. The needle remained in place at the injection site for 3 min before being slowly withdrawn over 2 min.

### Generation of adeno-associated virus (AAV: serotype 2/6) vectors

Human 4R0N cDNAs encoding wild-type and mutant P301S tau were each cloned into a pAAV shuttle vector harboring the AAV serotype 2 inverted terminal repeats (ITRs) and an expression cassette composed of the mouse PGK1 promoter, the human β-globin intron, a multiple cloning site (MCS) and the woodchuck hepatitis virus post-transcriptional regulatory element (WPRE) (modified from Stratagene's pAAV-MCS vector using standard cloning procedures). Both tau inserts were generated by PCR from previously described pHA/Tau vectors (Goedert et al., 1989) using the following primers: Forward 5′ CGA GTT CTC TCG AGG CCG CCA CCA TGG CTG AGC CCC GCC 3′ and Reverse 5′ CGA GTA CTA GAT CTT CAC AAA CCC TGC TTG GC 3′. PCR products were digested by XhoI and BglII and subcloned into the MCS of pAAV shuttle vector (XhoI/BglII) to generate pAAV-PGK-TauWT-WPRE and pAAV-PGK-Tau P301S-WPRE vectors.

### Production and titration of pseudotyped recombinant AAV2/6 vectors

Recombinant AAV2/6 vectors were produced and purified as previously described (Dusonchet et al., 2009). Briefly, AAV particles were generated by transient co-transfection of 293-AAV cells with pAAV shuttle vectors (bearing AAV2 ITRs) and a pDP6 helper plasmid (coding for AAV6 capsids) (Grimm et al., 2003) for 48 h. After collection, cells were lysed by three consecutive freeze/thaw cycles and AAV particles were purified on iodixanol density gradient followed by heparin affinity chromatography. Finally, AAV particles were eluted in sterile PBS and stored at − 80 °C until use. Following purification each batch was subjected to SDS-PAGE followed by a Coomassie staining to assess AAV purity. Infectious titers were determined as described previously (Dusonchet et al., 2009) and expressed in transducing units (TU)/ml. For the present study, the following vectors were produced: AAV-TurboGFP (fpmax): 2.8 × 10^10^ TU/ml; AAV-TauWT: 1.1 × 10^10^ TU/ml; and AAV-P301S: 1.7 × 10^10^ TU/ml.

### Stereotaxic injection of AAV vectors

AAV vectors encoding human wild-type tau (AAV-TauWT) or mutant P301S tau (AAV-P301S) were used to generate a local tauopathy in the mouse PRh. AAV-TurboGFP (fpmax) was used as a control vector. To inject AAV2/6 vectors in the PRh, stereotaxic surgery was performed under isoflurane anesthesia in 8–9 weeks old male C57BL/6S mice (Harlan, UK). In order to cover a significant portion of the PRh, the vector suspension was bilaterally injected at 4 sites (2 sites per hemisphere, each site injected with 0.5 μl at a rate of 0.2 μl/min) using a microsyringe pump controller (Micro4, World Precision Instruments). The stereotaxic coordinates for the PRh used in this study were 1. AP: − 1.8 mm; L: ± 4.2 mm; V: − 4.0 mm and 2. AP: − 3.2 mm; L: ± 4.1 mm; V: − 3.8 mm. The needle remained in place at the injection site for 3 min before slow withdrawn over a period of 2 min. The total dose of vector injected in each animal was 1 × 10^7^ TU. Animals were subjected to spontaneous OR task at 1 month and 2 months post-injection of the vector. Number of mice: AAV-fpmax *n* = 12, AAV-TauWT *n* = 13, and AAV-P301S *n* = 12.

### Immunohistochemistry

Animals were perfused transcardially with 4% paraformaldehyde (PFA) in 0.1 M PBS, pH 7.4. Brains were quickly dissected out and post-fixed overnight in 4% PFA at 4 °C, and cryoprotected in 30% sucrose in 0.1 M PBS for at least 24 h at 4 °C. Coronal brain sections (30 μm) were cut on a Leica SM2400 microtome (Leica Microsystems, Bucks, UK) and stored at 4 °C in 0.1 M PBS containing 0.1% sodium azide. A monoclonal antibody NeuN (1:400, Millipore) was used for neuronal cell counting. AT8 (1:1000, Innogenetics) and PHF1 (1:500, a gift of Dr Peter Davies) were used to detect hyperphosphorylated forms of tau. The biotinylated *Wisteria floribunda* agglutinin (WFA, 1:400, Sigma-Aldrich) was used to visualize PNNs. Labeling was performed by first rinsing tissue with 0.1 M phosphate buffered saline (PBS) followed by quenching of endogenous peroxidase activity with 10% methanol, 3% H_2_O_2_, and 0.1% Triton X-100 in PBS (PBS-T) for 20 min at room temperature (RT). Sections were rinsed three times in PBS-T and were subsequently blocked with 5% normal goat serum (NGS) or normal horse serum (NHS), in PBS-T for 1 h at RT. Sections were then incubated overnight at 4 °C with the primary antibody diluted in PBS-T. Following 3 washes in PBS they were incubated for 2 h at RT with the appropriate biotinylated secondary antibody (Vectastain; Vector Laboratories) diluted 1:1000 in PBS-T. The immunostaining was visualized with an avidin–biotin system (Vectastain; Vector Laboratories) and 3′,3′diaminobenzidine as the chromogen (DAB kit; Vector Laboratories). Sections were then mounted on glass slides, examined using a light microscope and photographed using a digital camera (Leica DM6000 Microsystems).

### Electrophysiology

Animals were anesthetized with isoflurane and decapitated. The brain was rapidly removed and placed in ice-cold cutting solution bubbled with 95% O_2_/5% CO_2_ containing the following (in mM): 132.8 NaCl, 3.1 KCl, 1 CaCl_2_, 2 MgCl_2_, 1 K_2_HPO_4_, 4 NaHCO_3_, 5 d-glucose, 0.01 glycine, 1 ascorbic acid, 0.5 myoinositol, 2 pyruvate, and 10 HEPES, adjusted to pH 7.35. A midsagittal section was made and the rostral part of one hemisphere was cut at 45° to the dorsoventral axis (Cho et al., 2000). The cerebellum was removed from the brain with a further caudal cut along the dorsoventral axis. The hemisphere was glued by its rostral end to a Vibratome stage (VT 1000S; Leica). Slices (350 μm) of PRh were taken in the region − 2.5 mm to − 4.0 mm rostral from bregma. Slices were stored submerged in bubbled, artificial CSF (20–25 °C, same composition as cutting solution, except 2 mM CaCl_2_, 1 mM MgCl_2_) for 2 h before the onset of recordings. A single slice was placed in an interface recording chamber superfused by artificial CSF (30 °C, flow rate 2 ml/min). Evoked field EPSPs (fEPSP) were recorded from layers II/III from directly below the rhinal sulcus (area 35). A stimulation electrode was placed in layer II/III on the temporal side (0.5 mm, area 36) of the recording electrode. Stimuli (0.1 ms duration) were delivered to the stimulation electrode at 0.1 Hz. Input/output curves were produced with stimulation intensities from 50 to 500 μA in steps of 50 μA. For monitoring baseline synaptic transmission before LTD induction, fEPSPs were reduced to 50–60% of the maximum amplitude and recorded for at least 30 min or until responses were stable (< 20% amplitude change over 30 min), (Number of mice for the study: WT P-nase *n* = 11, P301S P-nase *n* = 11, P301S ChABC *n* = 11). For LTD induction, 900 stimulus pairs (1 ms each, 20 ms). Subsequently, fEPSPs elicited by 0.1 Hz stimulation were recorded for further 60 min (number of mice for the study: WT P-nase *n* = 7, P301S P-nase *n* = 7, P301S ChABC *n* = 6). Field potentials were amplified with a CyberAmp 320 (Molecular Devices), recorded and analyzed with custom-made software written in LabView (National Instruments). For offline LTD analysis, fEPSPs were averaged across 1 min and the peak amplitude of the mean fEPSP was expressed relative to the preconditioning baseline. Group means were statistically compared using either paired or unpaired *t* tests or repeated-measures (RM) ANOVA, followed by tests of simple main effects, if applicable.

### Stereology

For stereological counting of neurons, pathological tau or PNN-positive cells, brain sections were stained with NeuN, AT8 or WFA, respectively. Immunolabeling of each antibody was revealed using 3,3′-diaminobenzidine tetrahydrochloride (DAB) and coverslips were applied using DPX mounting medium. Stereology was performed using an Olympus microscope with the stereological software StereoInvestigator (MBF Bioscience). A total number of 5–6 sections per animal (*n* = 5–6 mice for each group), taken every 12th brain sections were used for immunohistochemistry. The whole brain section was first highlighted at 4× objective and PRh contour was outlined. Sample counting windows were identified using the computer software. The number of sample counting windows was normally between 15  and 20. The number of NeuN, AT8 or WFA-positive cells in each window was counted at 40× magnification. The counting frame and the counting grid were set by the operator; 40 μm × 40 μm and 200 μm × 200 μm, respectively. Movements among counting frames of each grid were controlled by a motorized x–y microscope stage. Cells located inside the counting frame or touching its top or right borders only were counted. For the Z calibration, the tissue thickness at every counting frame was determined and the mean of sections thickness was taken into account for calculating the total number of cells. The total number of NeuN, AT8 or WFA-positive cells was calculated using the following equation:

$$N=\sum Q^{-}\cdot \frac{t}{h}\cdot \frac{1}{asf}\cdot \frac{1}{ssf}$$

asf: area sampling fraction
H: height of counting space
Q^−^: number of object
ssf: section sampling fraction
t: thickness of the section.

### Sarkosyl insoluble tau extraction

P-nase or ChABC was stereotaxically injected into C57BL/6S wild-type and P301S mice as described in the ChABC injections section. After 2 weeks from the injection, the mice were culled by cervical dislocation and brains were excised for sarkosyl-insoluble tau preparation, which was performed as previously described (Allen et al., 2002; Delobel et al., 2008) with a slight modification. Brain tissues containing mouse PRh were homogenized in ice with the electronic tissue grinder (IKA® ULTRA-TURRAX® dispersers, Germany) in 10-fold volume of A68 buffer (10 mM Tris–HCl pH 7.4, 0.8 M NaCl, 1 Mm EGTA and 10% sucrose) containing protease inhibitor cocktail (Roche). The homogenates were centrifuged at 2000 *g* for 10 min at 4 °C to remove membranes. Aliquot of the supernatant was used to quantify proteins. Samples were centrifuged again at 18,000 *g* for 30 min at 4 °C and then supernatant was collected into a new tube. The pellet was resuspended in 5-fold volume of A68 buffer by vortexing and was subjected to a second centrifugation at 18,000 *g* for 30 min. The first and second supernatants were pooled and then incubated with 1% N-Lauryl-Sarkosyl for 1 h at RT and subjected to ultracentrifugation at 70,000 *g* for 1 h at 4 °C (Optima™ Max Ultracentrifuge, Beckman Coulter, UK). The pellet was resuspended in Laemmli sample buffer (Sigma-Aldrich) and denatured for 15 min at 95 °C and loaded in the gel. The remaining amount was stored at − 20 °C until further use. (Number of mice for the study: Control: P-nase *n* = 2, ChABC *n* = 2; P301S: P-nase *n* = 4, ChABC *n* = 4).

### Protein quantification

Total protein concentration of brain homogenate was measured using the bicinchoninic-acid (BCA) assay (Pierce, Thermo Scientific, UK). Absorbance was read at 562 nm using a BioTek μQuant microplate reader. Protein concentration was determined by calculation from the standard curve using Excel software 2007 (Microsoft).

### Western blot analysis of sarkosyl-insoluble tau

Proteins were separated by electrophoresis on NuPAGE® Bis–Tris precast gel (Novex®, Life Technologies™) at 120 V for 90 min, and then transferred to PVDF membranes (Millipore Corporation) at 30 mA for 150 min. Non-specific background of membranes was blocked with incubation for 1 h at RT in TBS-T (10 mM Tris/HCl pH 7.8, 100 mM NaCl, 0.05% Tween 20) plus 5% (w/v) non-fat milk, and then washed three times with TBS-T. Membranes were incubated with rabbit anti-human tau polyclonal antibody (1:2000, DAKO), phosphorylation dependent anti-tau monoclonal antibody AT8 (1:1000, Innogenetics) or anti-β-actin antibody (1:5000, Sigma-Aldrich) overnight at 4 °C and then washed three times with TBS-T. This was followed by incubation with donkey anti-rabbit or sheep anti-mouse IgG-horseradish peroxidase (1:5000, GE Healthcare UK Ltd.) for 2 h at RT. This was followed by three washes with TBS-T. All the antibody dilutions were prepared in 1% non-fat milk in TBS-T and the duration of each wash step was 10 min. The proteins were detected by enhanced chemiluminescence (Pierce® ECL Western blotting substrate, Thermo Scientific, UK) and recorded by *chemiluminescence imaging system* (UVITEC Cambridge, UK).

## Results

### Progressive memory loss in Tg P301S mice

**Fig. 1:**
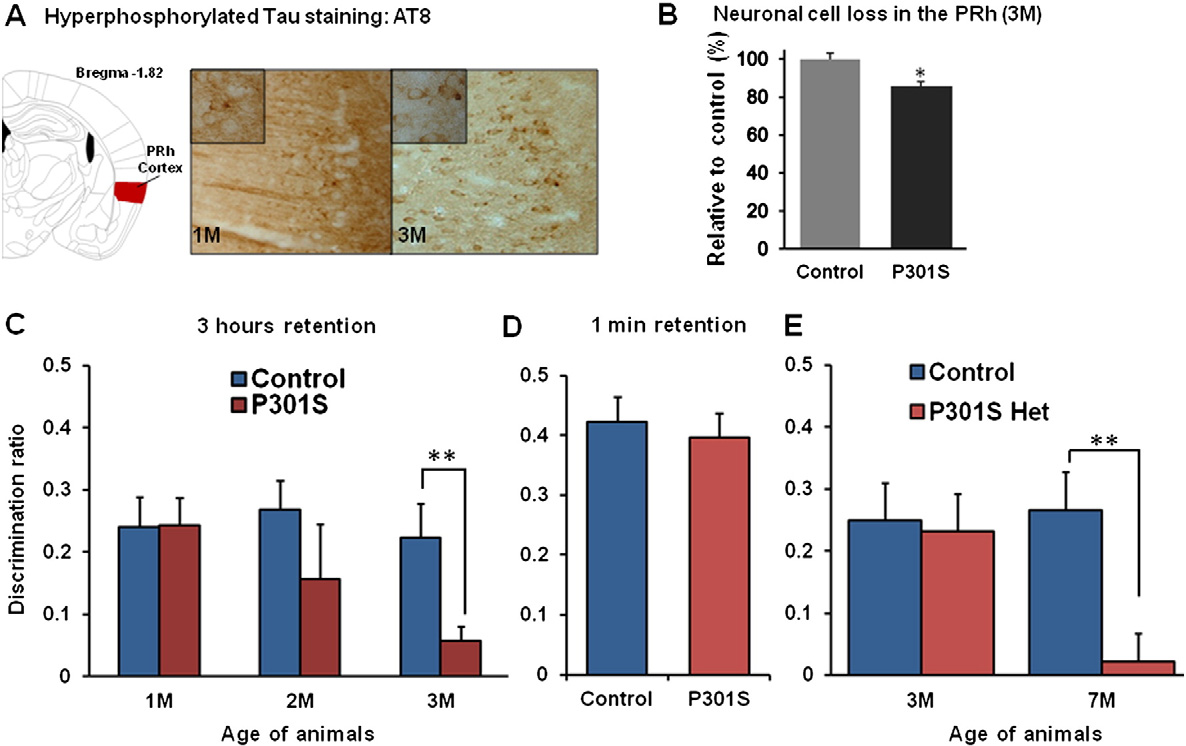
Temporal progression of OR memory deficit in Tg P301S mice. A. Brain sections from 1  to 3 month-old P301S mice were stained with anti-hyperphosphorylated tau antibody AT8. Tau hyperphosphorylation was visualized by AT8 in the PRh of P301S mice. The PRh at the bregma − 1.82 mm is shown in a coronal brain section image modified from brain atlas (Paxinos et al., 2001). In 3 month-old mice tau immunostaining appeared more localized in cell bodies compared to 1 month-old mice. No staining was present in control brain (data not shown) B. Brains from P301S mice and age-matched control (C57BL/6S) were immunostained with NeuN antibody. NeuN-positive neurons were counted by stereology quantification. The neuronal cell loss in the PRh was significant in 3 month-old P301S mice compared to controls (WT *n* = 5, P301S *n* = 6, *t*-test, *p < 0.05) C. P301S mice showed the spontaneous OR memory deficit in the 3 h retention paradigm. The discrimination ratio represented as (novel − familiar object exploration time) / total exploration time. The OR memory deficit was significant in 3 month-old P301S mice compared to control mice (WT *n* = 6, P301S *n* = 10, *t*-test, **p < 0.01). The progression of the deficit was age-dependent in P301S mice. No deficit was present in 1 month-old mice compared to controls (WT *n* = 7, P301S *n* = 9), while in 2 month-old mice a deficit was present but it did not reach significance (WT *n* = 5, P301S *n* = 7). D. Three month-old P301S and control mice were subjected to a 1 min delay between sample and choice phases to determine whether possible visual problem in the P301S mice could have affected the results. Both P301S and control mice showed a similar strength of OR memory after a 1 min delay. E. In order to verify specificity of the transgenic effect on the deficit in the spontaneous OR task 3 and 7 month-old littermate control and heterozygous P301S mice (P301S Het) were tested to determine their memory for 3 h retention in the spontaneous OR task. Both control and P301S Het mice showed identical OR memory at 3 months of age (WT *n* = 6, P301S Het *n* = 7) but P301S Het mice showed a significant deficit compared to their littermate controls at 7 months of age (WT *n* = 8, P301S Het *n* = 7, *t*-test, **p < 0.01). Data are presented as mean ± SEM.

We focused our study on the PRh as it has a well-established role in OR memory and is affected early in AD and tauopathies (Bartko et al., 2007; Mumby and Pinel, 1994; Winters et al., 2004). Tau pathology in the PRh of 1 and 3 month-old homozygous Tg P301S mice was characterized using monoclonal antibody AT8 which detects hyperphosphorylated tau (Fig. 1A). A low level of immunoreactivity with AT8 was already present at 1 month, with more intense staining at 3 months. At this latter age homozygous Tg P301S mice showed approximately 15% neuronal cell loss in the PRh compared to WT control mice (*p < 0.05) (Fig. 1B). In addition, many neurons had abnormal dystrophic dendritic morphology in Tg P301S mice. No difference in number of neurons was found between 1 month-old P301S mice and control mice (data not shown).

OR memory was compared between homozygous Tg P301S mice and age-matched controls at 1, 2 and 3 months old (Fig. 1C). Tg P301S mice showed a progressive deficit in their OR memory compared to controls. They had no abnormality at 1 month old but showed profound memory loss at three months (one-way ANOVA, *p < 0.05). The trend of memory deficit in Tg P301S mice started at 2 months and was significant at 3 months (WT 0.222 ± 0.055; *n* = 6 vs Tg P301S 0.0569 ± 0.023; *n* = 10, *t*-test, **p = 0.0018). WT control and Tg P301S mice explored the sample objects to a similar extent, indicating that there was no major difference in motivation between groups at different ages (Fig. S1). The deficit observed in 3 month-old P301S mice was not due to a general inability to discriminate objects or assess their familiarity/novelty, since both groups showed a similar level of discrimination for the novel object when the delay between sample and choice phase was minimal at 1 min (Fig. 1D). The study above compared homozygote transgenics with controls of the same strain; in order to confirm that the loss of memory was a transgene-related effect, P301S heterozygote (P301S Het) mice were compared to littermate controls. The P301S Het mice, with lower transgene expression, showed delayed tauopathy compared to the homozygotes. Three month-old P301S Het mice did not show impaired OR memory while a significant deficit in the OR memory was present at 7 months old compared to WT littermate controls (WT 0.265 ± 0.062; *n* = 8 vs P301S Het 0.022 ± 0.045; *n* = 7, *t*-test, **p = 0.0046). These results demonstrate that expression of the P301S tau mutation leads to a progressive memory deficit that depends on level of transgene expression.

OR memory was compared between homozygous Tg P301S mice and age-matched controls at 1, 2 and 3 months of age (Fig. 1C). Tg P301S mice showed a progressive deficit in their OR memory compared to controls. They had no abnormality at 1 month of age but showed profound memory loss at three months (one-way ANOVA, *p < 0.05). The trend of memory deficit in Tg P301S mice started at 2 months and was significant at 3 months (WT 0.222 ± 0.055; *n* = 6 vs Tg P301S 0.0569 ± 0.023; *n* = 10, *t*-test, **p = 0.0018). WT control and Tg P301S mice explored the sample objects to a similar extent, indicating that there was no major difference in motivation between groups at different ages (Fig. S1). The deficit observed in 3 month-old P301S mice was not due to a general inability to discriminate objects or assess their familiarity/novelty, since both groups showed a similar level of discrimination for the novel object when the delay between sample and choice phase was minimal at 1 min (Fig. 1D). The study above compared homozygote transgenics with controls of the same strain; in order to confirm that the loss of memory was a transgene-related effect, P301S heterozygote (P301S Het) mice were compared to littermate controls. The P301S Het mice, with lower transgene expression, showed delayed tauopathy compared to the homozygotes. Three month-old P301S Het mice did not show impaired OR memory while a significant deficit in the OR memory was present at 7 months of age compared to WT littermate controls (WT 0.265 ± 0.062; *n* = 8 vs P301S Het 0.022 ± 0.045; *n* = 7, *t*-test, **p = 0.0046). These results demonstrate that expression of the P301S tau mutation leads to a progressive memory deficit that depends on level of transgene expression.

### Memory loss following focal AAV-P301S injection to the PRh

**Fig. 2:**
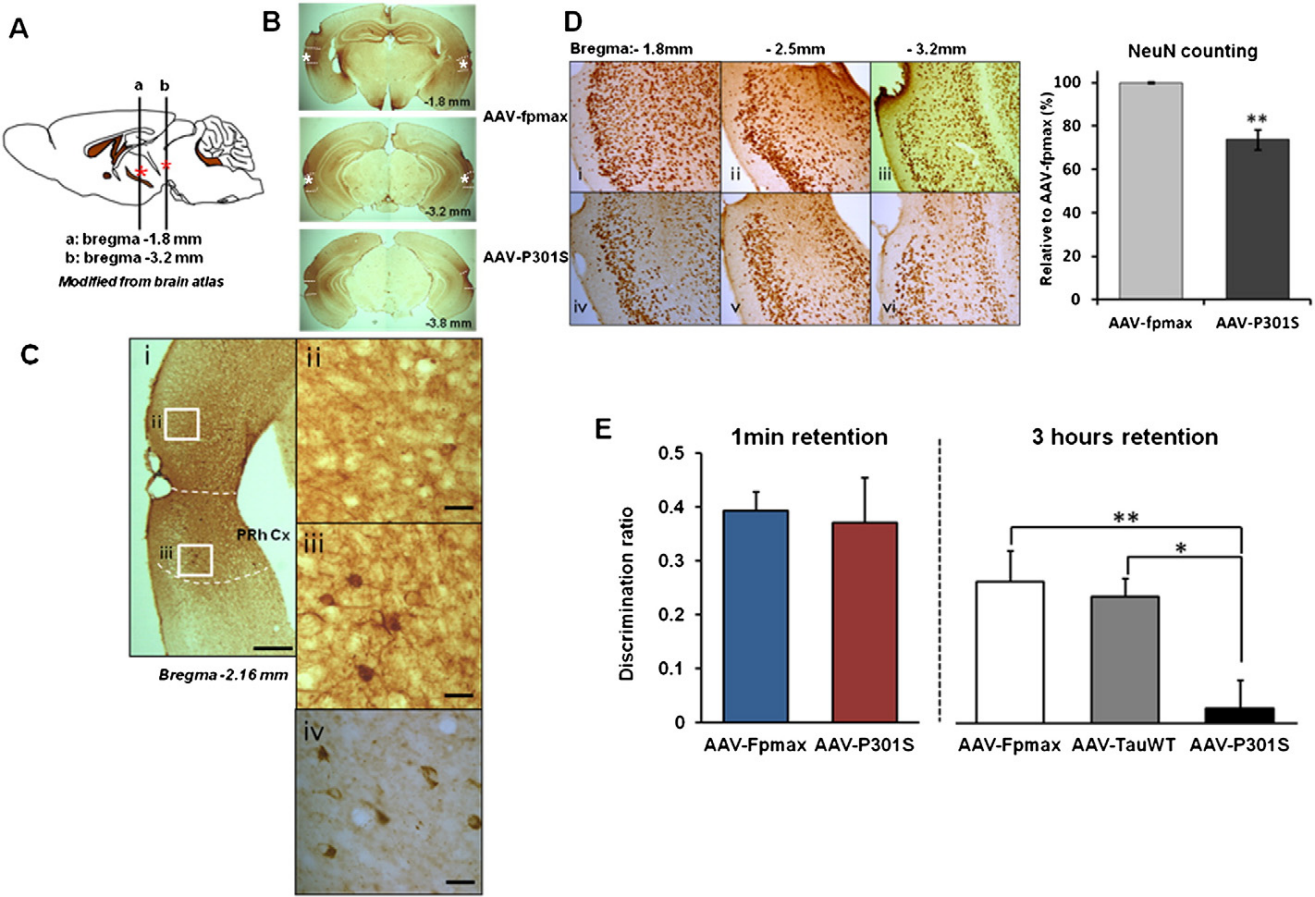
AAV-P301S induced neurodegenerative tauopathy and OR memory deficit. A. Schematic illustration for AAV-P301S injection sites. Asterisks indicated the injection site. B. Immunostaining with AT8 antibody 2 months after the bi-lateral injections of AAV-P301S into the PRh of C57BL/6S mice. Dashed lines indicate the area of the PRh. Asterisks indicate the injection site. C. More abundant tau pathology in the PRh cortex was present compared to adjacent cortices. i) Labeling with AT8 antibody revealed the tauopathy-induced lesion on the PRh at bregma − 2.16 mm. ii) Adjacent regions such as the entorhinal cortex were not affected as much as PRh. iii) AT8-positive neurons and dendrites were present in the PRh. iv) Hyperphosphorylated tau deposits were also detected by PHF1 antibody. Scale bar, i: 500 μm, and ii–iv: 30 μm D. Immunostaining with NeuN antibody showed a significant neuronal cell loss in the PRh. AAV-fpmax injected mice (i–iii) displayed less neuronal damage and significantly more neurons compared to AAV-P301S injected mice (iv–vi). This was confirmed by stereological quantification (AAV-fpmax *n* = 3, AAV-P301S *n* = 5, *t*-test, **p < 0.01). Data are presented as mean ± SEM. E. AAV-fpmax (*n* = 12), AAV-TauWT (*n* = 13) and AAV-P301S (*n* = 12) injected mice were tested in the spontaneous OR task for 1 min or 3 h of memory retention. There was no difference between AAV-fpmax and AAV-P301S groups after a 1 min delay while AAV-P301S injected mice showed a significant impairment in OR memory following a 3 h delay. ANOVA followed by Tukey post-hoc test, *p < 0.05, **p < 0.01.

In order to confirm that neurodegenerative tau pathology in the PRh of mice causes an OR memory deficit, human tau (4R0N) containing the P301S mutation was expressed in the PRh using an AAV vector as previously reported (Dassie et al., 2013). AAV-P301S was bilaterally injected into the PRh of adult C57BL/6S mice. Control groups were generated by injecting the same dose of AAV-fpmax (GFP control) and AAV-TauWT (wild-type tau) into age-matched animals. In animals injected with AAV-P301S, immunostaining for AT8 showed the presence of hyperphosphorylated tau around the injection sites at 2 months post-injection (Fig. 2A). The immunolabeling of AT8 was localized from rostral to caudal PRh, partially affecting adjacent regions of the entorhinal cortex and temporal association cortex (Fig. 2B). We also found PHF1 positive staining focused around the injection sites (Fig. 2C). 26% neuronal cell loss was present in the PRh in the AAV-P301S injected group compared to the control group injected with AAV-fpmax (Fig. 2D). Mice injected with AAV-fpmax, AAV-TauWT and AAV-P301S were assessed using the spontaneous OR task with 1 min or 3 h memory retention to detect PRh-related functional impairment (Fig. 2E). Fig. 2E shows OR memory at 2 months after injection of the vector: the AAV-P301S injected group had a significant deficit in 3 h memory retention compared to the other two groups (one-way ANOVA with Tukey post-hoc test, *p < 0.05, **p < 0.01). With a short delay between sample and choice phases showed normal discrimination, indicating that perirhinal tauopathy did not lead to a general inability to discriminate objects or assess their familiarity/novelty (Fig. 2E). Animals tested 1 month after injection of the AAV-P301S vector exhibited a reduced degree of tauopathy and OR memory deficit compared to mice tested 2 months after AAV-P301S injection (data not shown).

### ChABC injection restores memory in Tg P301S mice

**Fig. 3:**
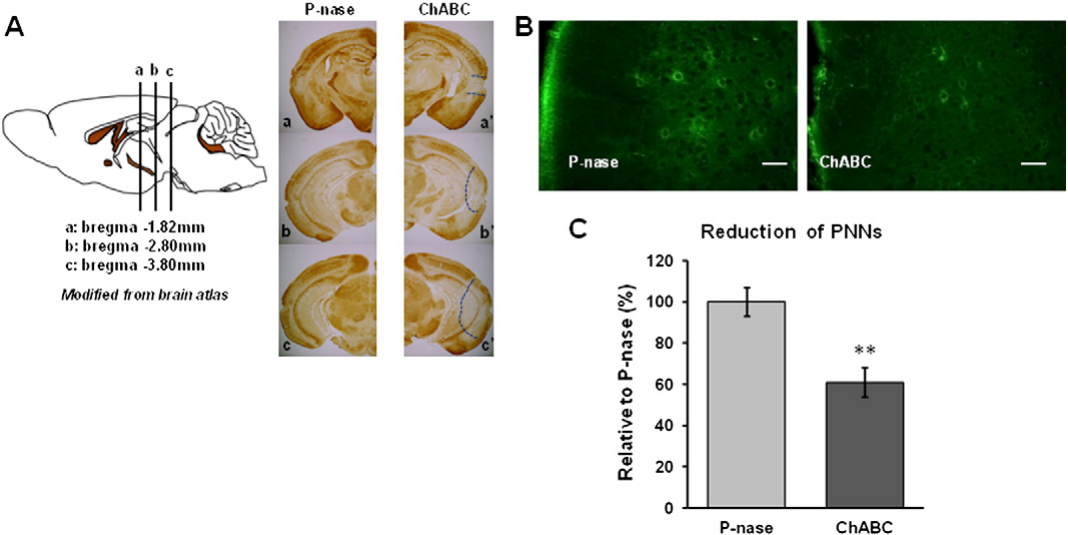
Removal of PNNs by ChABC treatment in Tg P301S mice. A. The sagittal brain atlas illustrates the injection levels of ChABC. Immunohistochemistry using WFA in P-nase (a–c) ChABC (a′–c′) injected Tg P301S mice at 2 weeks post-injection. It showed the extent of depletion of PNNs by ChABC treatment, indicated by the dotted line. B. Profiles of WFA-positive PNNs in the PRh of P301S mice at higher magnification following P-nase or ChABC injection. Scale bar, 50 μm C. Stereological analysis of WFA-positive neurons in the PRh of P301S mice following P-nase or ChABC injection. Data are presented as mean ± SEM. A significant decrease in WFA staining is caused by ChABC injection in Tg P301S mice (P-nase *n* = 6, ChABC *n* = 6, *t*-test, **p < 0.01).

Digestion of CSPGs in the adult CNS with ChABC reactivates plasticity and has a direct effect on memory. We hypothesized that treatment of the PRh with ChABC might recover the profound OR memory deficit described above. Various observations indicate that the main effect of ChABC is through its effect on PNNs. We therefore confirmed that PNNs were present in the PRh of Tg P301S mice and therefore available as a target for ChABC treatment (Fig. 3). PNNs were visualized with WFA lectin, which binds to chondroitin sulphate chains, the target of ChABC. The number and intensity of PNNs was similar in the P301S homozygotes at 3 months compared to age-matched WT control mice (Fig. S2).

Digestion of CSPGs in the adult CNS with ChABC reactivates plasticity and has a direct effect on memory. We hypothesized that treatment of the PRh with ChABC might recover the profound OR memory deficit described above. Various observations indicate that the main effect of ChABC is through its effect on PNNs. We therefore confirmed that PNNs were present in the PRh of Tg P301S mice and therefore available as a target for ChABC treatment (Fig. 3). PNNs were visualized with WFA lectin, which binds to chondroitin sulphate chains, the target of ChABC. The number and intensity of PNNs was similar in the P301S homozygotes at 3 months compared to age-matched WT control mice (Fig. S2).

**Fig. 4:**
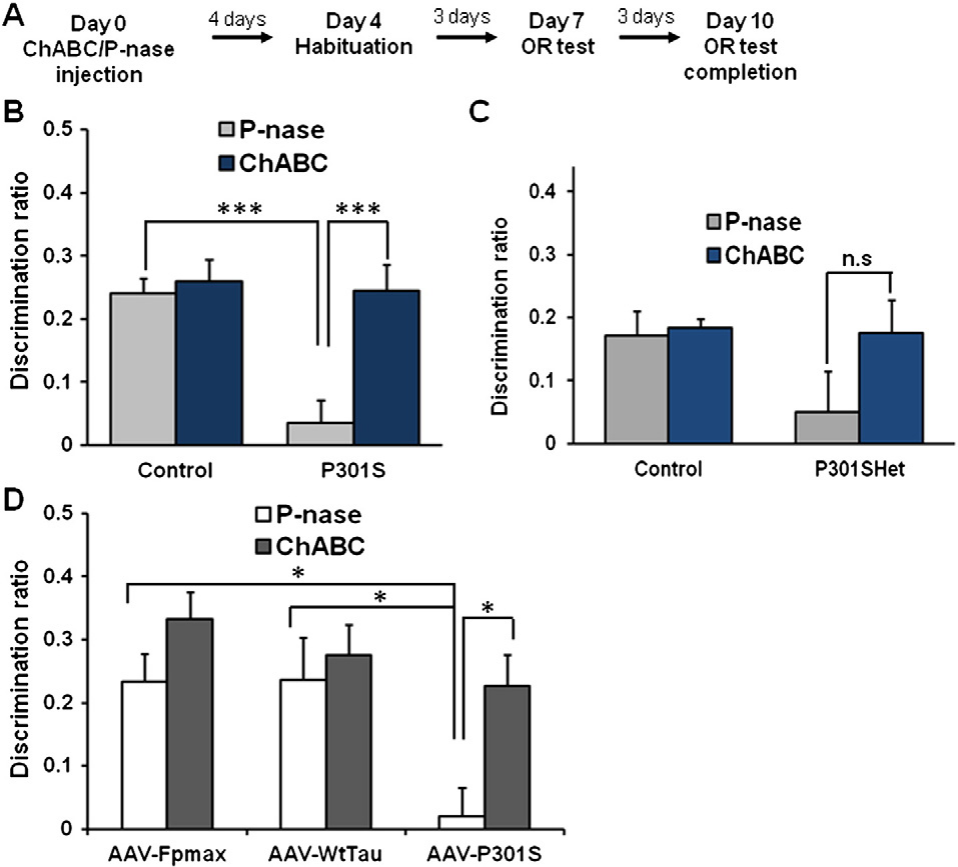
OR memory recovery after ChABC treatment in Tg P301S mice and AAV-P301S injected mice. A. Experimental protocol for the OR test after P-nase or ChABC injection. B. Three month-old homozygous P301S and age-matched control mice were injected with P-nase or ChABC. ChABC injected P301S mice showed a significant improvement in the OR memory compared to P-nase injected mice in spontaneous OR paradigm with a 3 h delay (P-nase *n* = 16, ChABC *n* = 13, *t*-test, ***p < 0.001) whereas control mice were not affected by ChABC (P-nase *n* = 7, ChABC *n* = 5) C. Seven month-old P301S Het and the age-matched littermate control mice were injected either with P-nase or ChABC. Spontaneous OR test with a 3 h delay was applied for testing OR memory after treatment. Similar pattern to that shown in B was observed. Although due to the variability in P301S Het mice, particularly those treated with P-nase, the results did not reach significance. Control: P-nase *n* = 4, ChABC *n* = 3; P301S: P-nase *n* = 4, ChABC *n* = 4 D. Mice from all AAV injected groups were tested by the spontaneous OR task with a 3 h delay-paradigm following P-nase or ChABC treatment to the PRh cortex. The OR deficit exhibited in the AAV-P301S injected mice (ANOVA with Tukey post-hoc, *p < 0.05) was significantly improved by ChABC treatment (*t*-test, *p < 0.05). AAV-fpmax: P-nase *n* = 6, ChABC *n* = 6; AAV-TauWT: P-nase *n* = 6, ChABC *n* = 6; AAV-P301S: P-nase *n* = 6, ChABC *n* = 6.

In order to attenuate the PNNs in the PRh, the CSPG-degrading enzyme ChABC was bilaterally injected at three different sites into 3 month-old Tg P301S mice or 7 month-old Tg P301S Het mice. The extent of PNN digestion was visualized by WFA staining two weeks after treatment (Fig. 3A). At this time there was an obvious reduction in the intensity of WFA staining in the injected area, indicating successful ChABC digestion in the PRh with some extension to the adjacent regions including the entorhinal cortex, temporal associated cortex and caudal hippocampus (Figs. 3A–B). We also counted WFA positive neurons in the PRh, finding a 39% reduction two weeks after ChABC injections compared to animals injected with the control bacterial enzyme P-nase (P-nase *n* = 6, ChABC *n* = 6, *t*-test, **p < 0.01) (Figs. 3B–C). Seven days after ChABC or P-nase treatment animals were tested for OR memory (Fig. 4A). The impaired OR memory found in 3 month-old P301S mice at a 3 h delay was restored to a level indistinguishable from that of control animals after ChABC treatment while animals treated with control enzyme P-nase showed the same deficit as untreated animals (P301S/P-nase; *n* = 16, P301S/ChABC; *n* = 13, *t*-test, ***p = 0.0003) (Fig. 4B). Age-matched C57BL/6S control mice showed minimal change in the OR memory between P-nase and ChABC treated groups at 3 h retention (Control/P-nase; *n* = 7, Control/ChABC; *n* = 5, *t*-test, *p* = 0.6352), as in our previous study (Romberg et al., 2013) (Fig. 4B). Seven month-old Tg P301S Het mice which have a similar deficit in OR memory to 3 month-old Tg P301S homozygous mice showed a similar restoration trend of OR memory in ChABC treated animals (P301S Het/P-nase; *n* = 4, P301S Het/ChABC; *n* = 3, *t*-test, *p* = 0.2155), with no effect in WT littermate controls (Control/P-nase; *n* = 4, Control/ChABC; *n* = 4, *t*-test, *p* = 0.7567) (Fig. 4C). Although due to the variability in P301S Het mice, particularly those treated with P-nase, the results did not reach significance.

### ChABC injection restores memory in AAV-P301S model

We next asked whether digestion of PNNs with ChABC can restore OR memory in the focal AAV tauopathy model. Adult animals were injected with either AAV-fpmax, AAV-TauWT or AAV-P301S at 8–9 weeks of age. At 2 months post-injection, as described above, AAV-P301S injected mice showed a profound impairment in OR memory, while the control AAV- fpmax or AAV-TauWT injected mice displayed normal OR memory in the 3 h delay paradigm (Fig. 2E). Then they were bilaterally injected with either P-nase or ChABC using the same injection paradigm as for Tg P301S mice (Fig. 3A), followed by OR testing on the schedule described in Fig. 4A. ChABC injected AAV-P301S mice had significantly improved OR memory compared to P-nase injected AAV-P301S mice in the 3 h delay paradigm (*t*-test, *p < 0.05) (Fig. 4D).

### Memory deficit returns 5 weeks after ChABC treatment

**Fig. 5:**
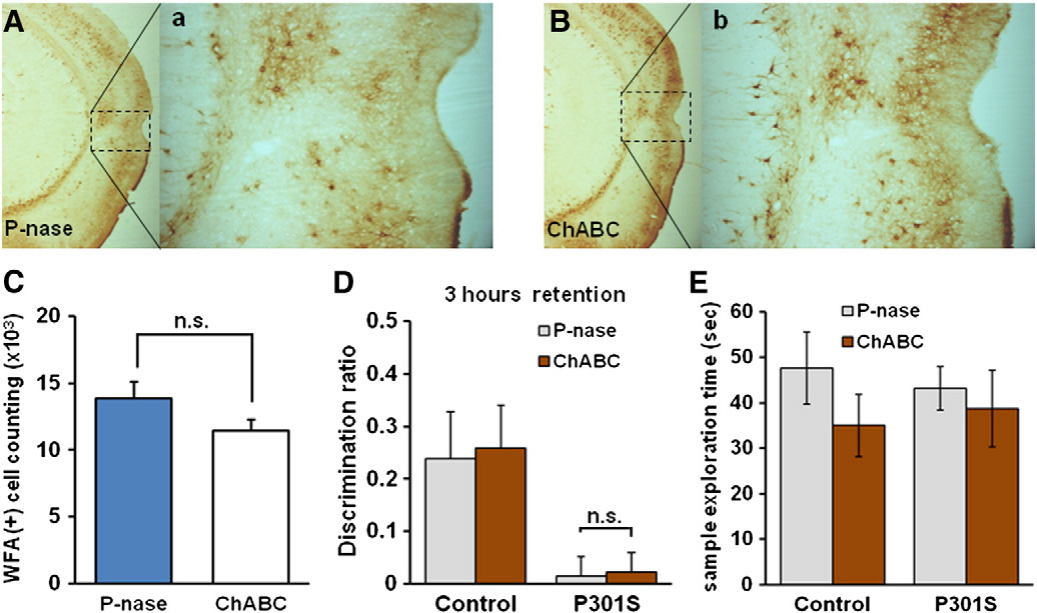
OR memory deficit returns 5 weeks after ChABC treatment in Tg P301S mice PNNs were visualized by WFA staining in P-nase (A) or ChABC (B) injected mice 5 weeks post-injection. The PNNs present in the PRh of P-nase (a) or ChABC (b) injected mice were shown at higher magnification. C. Stereological quantification of WFA-positive cells in the PRh of P301S mice following P-nase (*n* = 5) or ChABC (*n* = 5) injection. There was no significant difference between groups (*t*-test, n.s: not significant, *p* = 0.072). D. ChABC and P-nase treated P301S mice showed similar level of deficit 5 weeks following ChABC injection. At this time point, ChABC treatment was no longer effective in restoring the OR memory in Tg P301S mice (P-nase *n* = 7, ChABC *n* = 6, *t*-test, n.s, *p* = 0.91). E. The exploration time for sample phase of control and P301S mice at 5 weeks after ChABC injection, indicating that each group presented comparable extent of motivation and that lack of motivation was not interfering with the results. Control; P-nase *n* = 6, ChABC *n* = 5, P301S; P-nase *n* = 7, ChABC *n* = 6.

Our data show that ChABC to the PRh can reverse OR memory impairment in two different models of tauopathy-induced memory loss. As shown in the schedule in Fig. 4A, the memory tests were performed 7–10 days after ChABC injection. Turnover of stable extracellular structures such as PNNs is relatively slow, so we wished to know for how long memory would be restored after a single ChABC injection. Staining for PNNs in Tg P301S mice after P-nase or ChABC injection showed that PNN intensity and number had almost returned to normal by 5 weeks (*n* = 5 per group, *t*-test, *p* = 0.072) (Figs. 5A–C). We therefore measured OR memory at this time. By 5 weeks after ChABC treatment P301S mice no longer showed the improved OR memory that was seen at 10 days (P301S/P-nase vs P301S/ChABC, *t*-test, *p* = 0.91) (Fig. 5D). Motivation measure by exploration time showed no difference between groups (Control/ChABC vs P301S/ChABC, *t*-test, *p* = 0.642) (Fig. 5E).

### ChABC restores synaptic transmission

**Fig. 6:**
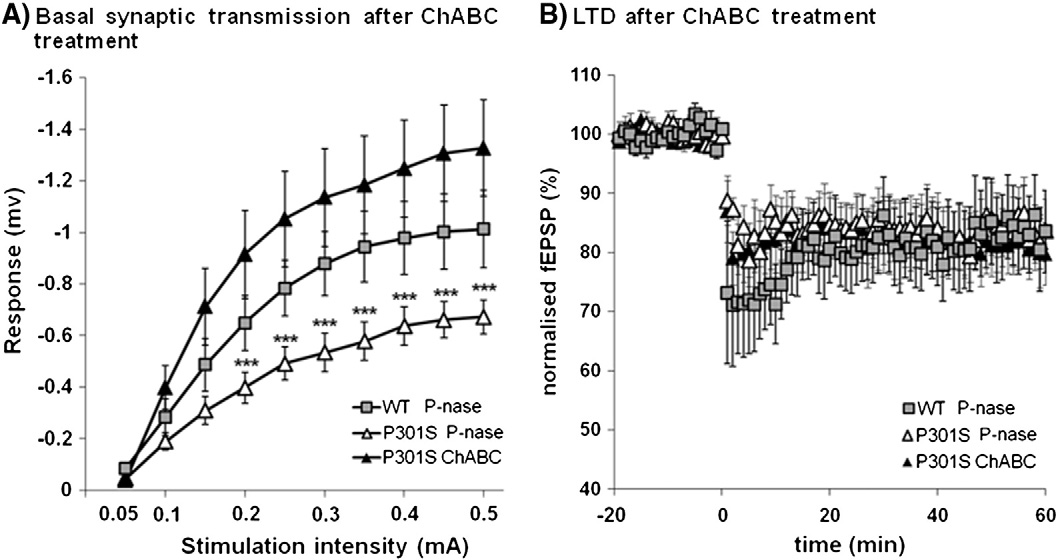
ChABC treatment altered synaptic transmission in the PRh of Tg P301S mice. A. Layer II/III fEPSP amplitudes after stimulation of temporal layer II/III input with different stimulus intensities. WT P-nase *n* = 11, P301S P-nase *n* = 11, P301S ChABC *n* = 11. Two-way RM ANOVA: there is a statistically significant interaction between group and intensity. Data are presented as mean ± SEM. ***p < 0.001 B. LTD induced by low-frequency stimulation is not different between P-nase injected WT control and P301S mice. Moreover, ChABC treatment to the PRh did not alter LTD in P301S mice. WT P-nase *n* = 7, P301S P-nase *n* = 7, P301S ChABC *n* = 6.

OR memory requires normal synaptic transmission in the PRh and has been shown to involve long-term depression (LTD) in neuronal responsiveness in this area (Brown et al., 1987; Griffiths et al., 2008; Xiang and Brown, 1998; Zhu et al., 1996). In our previous study on the effects of PNN depletion in the PRh of WT mice, we demonstrated an increase in synaptic transmission and LTD compared to controls (Romberg et al., 2013). To investigate the physiological changes in PRh when affected by tau pathology, and the effects of ChABC, we measured PRh synaptic transmission and LTD in coronal slices from 3 month-old Tg P301S mice with and without ChABC treatment, and from normal age-matched controls (Fig. 6). Stimulation of temporal cortex input into layer II/III of the PRh with different stimulus intensities revealed that basal fEPSPs were significantly diminished below control levels in Tg P301S mice. fEPSP amplitude was restored to control levels following ChABC treatment showing a significant trend to surpass normal fEPSP values (Fig. 6A). There was a significant interaction between group and intensity (RM ANOVA, *p* < 0.001, *F* = 4.335), and post-hoc analysis showed differences between the stimulus intensity vs response curves. LTD induction was unaffected in Tg P301S animals although it was impervious to ChABC treatment (Fig. 6B). These results show a considerable impairment in synaptic transmission in Tg P301S mice. As in our previous work, the removal of PNNs greatly increased the neuronal synaptic transmission in our link protein Crtl1 KO mice and ChABC-injected normal animals (Romberg et al., 2013).

### Effect of ChABC injection on hyperphosphorylated tau in Tg P301S mice

**Fig. 7:**
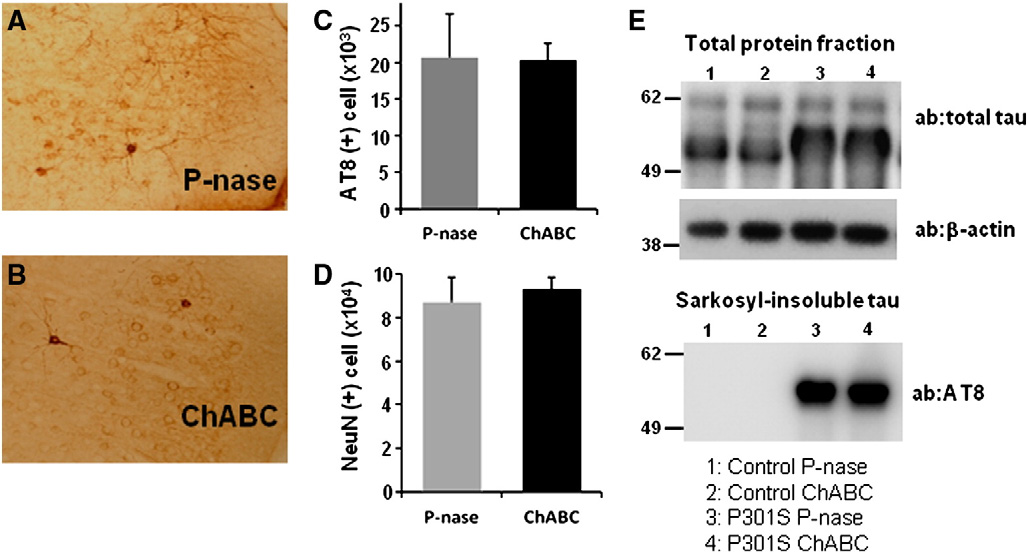
Tauopathy after ChABC treatment in Tg P301S mice. Hyperphosphorylated tau was visualized by AT8 antibody immunostaining in the PRh of P-nase (A) and ChABC (B) injected mice at 2 weeks post-injection. AT8 (C) or NeuN-positive cells (D) were stereologically quantified in the PRh cortex of P301S mice (P-nase *n* = 5, ChABC *n* = 5). No significant influence of ChABC treatment on AT8 staining or neuronal cell number was present in the Tg P301S mice. E. The level of total tau protein and sarkosyl-insoluble tau in the PRh treated with either P-nase or ChABC was analyzed by Western blotting using anti-human tau antibody. No difference was present between the two groups. Western blotting with AT8 antibody showed the similar level of sarkosyl-insoluble hyperphosphorylated tau in the P301S mouse PRh treated with either P-nase or ChABC, indicating that the treatment did not affect insoluble tau pathology. Control; P-nase *n* = 2, ChABC *n* = 2, P301S; P-nase *n* = 4, ChABC *n* = 4.

The above data show that ChABC treatment reverses the OR memory impairment in P301S mice. This functional restoration could be due to removal of PNNs and consequent promotion of plasticity and synapse dynamics. However, the aggregation of normal and mutant tau is influenced by chondroitin sulphate and heparan sulphate proteoglycans (Goedert et al., 1996), and it is therefore possible that ChABC treatment might influence on tau aggregation in Tg P301S mice. The profiles of pathological tau in Tg P301S mice after ChABC treatment to PRh were analyzed using the hyperphosphorylated tau specific antibody AT8, and compared to mice that received P-nase treatment (Figs. 7A and B). In the PRh, AT8 staining showed many neurons with various stages of tau aggregation, such as “ring”-like cytoplasmic accumulation and pre-tangle conformation. Stereological quantification of AT8-positive staining with all types of tau deposits showed no significant difference in the PRh of P301S mice 2 weeks after P-nase or ChABC injection (P-nase *n* = 5, ChABC *n* = 3, *t*-test, *p* = 0.9697) (Fig. 7C) or at 5 weeks after treatment (Fig. S3). In addition when cells manifesting “tangle”-like AT8-positive staining were selectively counted in the PRh there was no evidence that ChABC affected the pattern of tauopathy in P301S mice (data not shown). Moreover the number of NeuN positive neuronal cells in P301S mice was not affected by ChABC treatment at two weeks (*n* = 3/group, *t*-test, *p* = 0.6471) (Fig. 7D) and at five weeks (Fig. S3). We also analyzed tau levels and aggregation using a biochemical assay. Fresh frozen tissue from the areas injected with P-nase or ChABC from P301S or wild type control mice were subjected to sarkosyl extraction (Delobel et al., 2008), in order to enrich for aggregated tau. Western blot analyses of total protein fractions extracted from P-nase and ChABC treated control or P301S mice did not show any difference in the level of total tau (Fig. 7E). In the sarkosyl-insoluble fractions, containing mainly the aggregated proteins there was no significant change in the level of tau between P-nase and ChABC treated P301S mice (Fig. 7E). This trend was observed in four independent experiments using P301S mice. These data demonstrate that the progressive tauopathy and neurodegeneration seen in P301S mice is not significantly affected by ChABC treatment.

The above data show that ChABC treatment reverses the OR memory impairment in P301S mice. This functional restoration could be due to removal of PNNs and consequent promotion of plasticity and synapse dynamics. However, the aggregation of normal and mutant tau is influenced by chondroitin sulphate and heparan sulphate proteoglycans (Goedert et al., 1996), and it is therefore possible that ChABC treatment might influence tau aggregation in Tg P301S mice. The profiles of pathological tau in Tg P301S mice after ChABC treatment to PRh were analyzed using the hyperphosphorylated tau specific antibody AT8, and compared to mice that received P-nase treatment (Figs. 7A and B). In the PRh, AT8 staining showed many neurons with various stages of tau aggregation, such as “ring”-like cytoplasmic accumulation and pre-tangle conformation. Stereological quantification of AT8-positive staining with all types of tau deposits showed no significant difference in the PRh of P301S mice 2 weeks after P-nase or ChABC injection (P-nase *n* = 5, ChABC *n* = 3, *t*-test, *p* = 0.9697) (Fig. 7C) or at 5 weeks after treatment (Fig. S3). In addition when cells manifesting “tangle”-like AT8-positive staining were selectively counted in the PRh there was no evidence that ChABC affected the pattern of tauopathy in P301S mice (data not shown). Moreover the number of NeuN positive neuronal cells in P301S mice was not affected by ChABC treatment at two weeks (*n* = 3/group, *t*-test, *p* = 0.6471) (Fig. 7D) and at five weeks (Fig. S3). We also analyzed tau levels and aggregation using a biochemical assay. Fresh frozen tissue from the areas injected with P-nase or ChABC from P301S or wild type control mice were subjected to sarkosyl extraction (Delobel et al., 2008), in order to enrich for aggregated tau. Western blot analyses of total protein fractions extracted from P-nase and ChABC treated control or P301S mice did not show any difference in the level of total tau (Fig. 7E). In the sarkosyl-insoluble fractions, containing mainly the aggregated protein there was no significant change in the level of tau between P-nase and ChABC treated P301S mice (Fig. 7E). This trend was observed in four independent experiments using P301S mice. These data demonstrate that the progressive tauopathy and neurodegeneration seen in P301S mice is not significantly affected by ChABC treatment.

## Discussion

The overall aim of these experiments was to ask whether promoting plasticity and enhancing memory through modification of PNNs could allow the CNS to recover memory function in the presence of damage caused by tauopathy. We first defined the time course of loss in spontaneous OR memory in both a transgenic mouse model of familial tauopathy, and a focal tauopathy model induced by injection of AAV containing a mutant tau cDNA. Selecting a time point at which there is profound memory loss for each model, we then demonstrated that this deficit can be corrected by digestion of PNNs with ChABC, and that electrophysiological measures of synaptic transmission can be restored. Finally, we examined the effect of ChABC treatment on tau pathology and found that neither the progressive tauopathy nor the neurodegeneration seen in P301S mice was affected by a single treatment with ChABC; the effect of the ChABC treatment is therefore through promoting plasticity and memory function rather than through disease modification. Previous work using ChABC to promote plasticity has shown recovery in function in focal lesions such as spinal cord injury and stroke (Bradbury et al., 2002; Soleman et al., 2012); the present work shows that functional restoration can also be achieved in the presence of the more distributed neuronal dysfunction and loss created by neurodegeneration.

### Tauopathy progression and memory loss

In the Tg P301S mice there is a steady progression of tau pathology with hyperphosphorylated tau becoming more insoluble and filamentous as can be seen by biochemical isolation and by electron microscopy (Allen et al., 2002). At between two and three months of age in Tg P301S mice many neurons start to develop dystrophic morphology, with abnormal dendrites and perinuclear tau accumulations. In the PRh 15% neuronal cell loss was present at 3 months of age in homozygous P301S mice. A similar set of events occurs after injection of AAV-P301S into the cortex, as reported previously (Dassie et al., 2013). However, progression is faster in the injection area, and 2 months after the AAV-P301S injection the pathology resembles that seen in the transgenic animals at around 5 months of age, with 26% loss of neurons in the region immediately adjacent to the injection sites. A similar time course of memory loss was seen in the water maze test following AAV-P301S injections to the entorhinal cortex in a previous paper (Dassie et al., 2013). We tested OR memory at various ages in homozygous Tg P301S animals, finding almost normal memory at 1 month, around 50% reduction at 2 months, and a complete memory loss at 3 months. There was an equally profound loss of memory in the AAV-P301S animals at 2 months after vector injection. However, animals were able to learn to recognize objects, because when placed in the Y-maze 1 min after first exposure to an object they were able to recognize it as familiar and discriminate it from the novel object. On the other hand memory retention at 1 and 3 h after first exposure to the sample objects was eliminated. At this stage of pathology many neurons are dystrophic but few have died, so the functional loss must be mainly due to neuronal and synaptic dysfunction.

### Restoration of memory

The hypothesis that PNN digestion might restore memory was based on two previous studies. Gogolla et al. digested PNNs in the amygdala, showing that erasure of fear conditioning memory could now be achieved as in juveniles, changing the pattern from untreated adult animals where memory is resistant to extinction (Gogolla et al., 2009). Similarly, it has been recently reported that the enzymatic removal of ECM including PNNs in auditory cortex enhanced reversal learning memory without erasing the pre-existing memories (Happel et al., 2014). Previous work from our laboratories examined OR memory in animals with attenuated PNNs due to the Crtl1/Hapln1 gene deletion (Crtl1 KO) in the brain resulting in a link protein knock-out (a component of PNNs), and in normal animals, in which PNNs in the PRh were digested with ChABC. OR memories in mice usually decay by 12 h, but Crtl1 KO and ChABC-injected normal animals retained their memories for over 48 h (Romberg et al., 2013). Whereas that study used normal mice, the present study shows that even in mice with brains extensively damaged by neurodegeneration, digestion of PNNs can restore OR memory. This effect is possibly due to “activation” of neurons without tau pathology since we have observed that PNNs are mainly present around parvalbumin-positive interneurons without neurofibrillary tangles (SY unpublished observations). The memory enhancing effect in the mice with tauopathy was completely diminished by 5 weeks after ChABC treatment. However this effect might be prolonged if CSPG digestion is achieved for longer time for example by using ChABC gene delivery via lentiviral vector (Bartus et al., 2014).

### PNNs and memory

Although our study is focused on the effect of ChABC on PNNs they only contain 2% of brain CSPGs (Deepa et al., 2006). Therefore other targets of the enzyme cannot be excluded. However, the identification of PNNs as the target of ChABC digestion comes from the finding that animals lacking PNNs due to deletion of a link protein in the CNS have identical changes in plasticity to those treated with ChABC, and that further treatment of Crtl1 KO animals with ChABC (removing the remaining 98% of glycans) has no additional effect on memory (Carulli et al., 2010).

The mechanism by which PNNs might affect memory is not fully understood. PNNs are found particularly around parvalbumin-positive GABAergic interneurons. These cells control the initiation and termination of the developmental critical periods, termination being dependent on the formation of PNNs (Beurdeley et al., 2012; Pizzorusso et al., 2002). Parvalbumin-positive interneurons are responsible for controlling overall levels of inhibition, generation of rhythms and network synchronization (Bartos et al., 2007; Le Magueresse and Monyer, 2013). Interference with the function of these cells by transgenic manipulation of glutamate receptors or expression of tetanus toxin leads to impaired memory, and affects gamma oscillations which provide a temporal structure for information processing, including memory formation (Korotkova et al., 2010; Murray et al., 2011). During memory acquisition or after ChABC treatment the level of inhibitory synaptic input to parvalbumin-positive interneurons increases; this is accompanied by a decrease in the level of parvalbumin and the level of the enzyme glutamate acid dehydroxylase (responsible for GABA production) suggesting a lower level of cortical inhibition (Donato et al., 2013). Furthermore, PNNs have several properties that could affect synaptic function. Brevican, a PNN component, binds AMPA receptors, restricting their mobility and affecting short-term plasticity (Frischknecht et al., 2009), with the neuronal activity-related pentraxin (NARP) as a probable scaffolding intermediate (Gu et al., 2013). PNNs bind to and present a variety of molecules, including Semaphorin 3A, which has marked effects on synapse dynamics. Interference with Sema3A/Neuropilin-1/PlexinA4 signaling during development results in lasting changes in cortical neuron basal dendrite patterning, arborization and spine formation (Fenstermaker et al., 2004; Tran et al., 2009; Yamashita et al., 2007), Sema3A affects synaptic transmission in hippocampal neurons (Bouzioukh et al., 2006; Sahay et al., 2005) and blocking Sema3A receptors partially restores ocular dominance plasticity to the cortex (Pizzorusso unpublished observations). CSPGs are a major component of the PNNs and they have inhibitory effects via the PTPσ receptor, which is widely expressed in the cortex (Shen et al., 2009). It is possible that the dense and stable matrix in which synapses are embedded affects their behavior and the possibility of forming new connections. The PNN also binds Otx2, which maintains the maturity of the structures (Beurdeley et al., 2012; Spatazza et al., 2013). In addition, digestion of CSPGs can promote sprouting of new connections and restoration of function despite large CNS lesions (Kwok et al., 2011). Some degree of rewiring of the cortex as seen after CNS lesions must be necessary to compensate for neuronal loss in neurodegenerative disease. Indeed, we saw evidence for effects on synaptic transmission in our study, which might be a consequence of compensational rewiring of neuronal activity. Cortical slices from Tg P301S mice showed a marked diminution of fEPSP size in response to temporal stimulation, but this measure of synaptic activity was restored to normal or above following ChABC treatment, suggesting that some of the mechanisms mentioned above affect synapse number and/or activity.

### Treatment of neurodegenerative disease

At present there are no treatments that modify disease progression sufficiently to halt memory decline in Alzheimer's disease or tauopathies in human patients. Symptomatic treatment with memory-enhancing compounds such as cholinesterase inhibitors is modestly effective at some stages of the disease. The effect of ChABC that we have demonstrated suggests that manipulation of PNNs might be an alternative way of restoring memory, at least temporarily, in neurodegenerative disease. In the two mouse models that we have presented, ChABC treatment restored memory in the presence of advanced pathology. The effect of ChABC on diffuse pathology is not restricted to tau pathology, because in a recent experiment ChABC was injected into the hippocampus of transgenic mice with an amyloidopathy model of neurodegenerative disease (APP/PSI mice), demonstrating restoration of fear memory and in increase in LTP (Vegh et al., 2014). However, ChABC itself is not a viable treatment for neurodegenerative diseases; it has to be injected directly into the CNS because it does not cross the brain–blood barrier, and as we show its effects last for less than five weeks, the time taken by degraded PNNs to be normalized again. However other compounds, currently under development, that affect the PNNs in other ways, through actions on the glycan chains of CSPGs or targeting other PNN components could be effective in improving memory in these devastating diseases with tau pathology. The open question is how long it would be possible to postpone the loss of CNS function for example in Alzheimer's disease by using a PNN-targeted treatment. The finding that we were able to restore normal memory in the presence of advanced tau pathology suggests that a significant delay might be achieved in memory decay in tauopathies.

The following are the supplementary data related to this article.

Fig. S1 Sample exploration time for Tg P301S mice at different ages.
Sample exploration times were measured in P301S mice and age-matched control mice at 1 M, 2 M and 3 M olds. No difference between P301S mice and control mice was found. Data are presented as mean ± SEM. 1 M: Control *n* = 7, P301S *n* = 9; 2 M: Control *n* = 5, P301S *n* = 7; 3 M: Control *n* = 6, P301S *n* = 10.

Fig. S2 PNNs are present in Tg P301S mice.
A. Brain slices of P301S mice were stained with biotin-WFA and AT8 antibodies. WFA-positive neurons in the PRh were visible in P301S mice. Green: WFA-positive, red: AT8-positive, blue: DAPI B. Stereological analysis of WFA-positive neurons in the PRh of control and P301S mice at 3 month old. Data are presented as mean ± SEM. Control *n* = 4 P301S *n* = 6.

Fig. S3 Tauopathy after 5 weeks of ChABC treatment in Tg P301S mice.
A. Hyperphosphorylated tau was visualized by AT8 antibody immunostaining in the PRh of P-nase and ChABC injected mice at 5 weeks post-injection. AT8 (B) or NeuN-positive cells (C) were stereologically quantified in the PRh cortex of P301S mice (P-nase *n* = 6, ChABC *n* = 6). No significant influence of ChABC treatment on AT8 staining or neuronal cell number was present in the Tg P301S mice. Data are presented as mean ± SEM.

Supplementary data to this article can be found online at http://dx.doi.org/10.1016/j.expneurol.2014.11.013.
