# Supplementary figures and images for: Perineuronal net digestion with chondroitinase restores memory in mice with tau pathology
Source: Exp Neurol. 2015 Mar;265:48–58. doi: 10.1016/j.expneurol.2014.11.013 (PMC4353684; doi:10.1016/j.expneurol.2014.11.013)

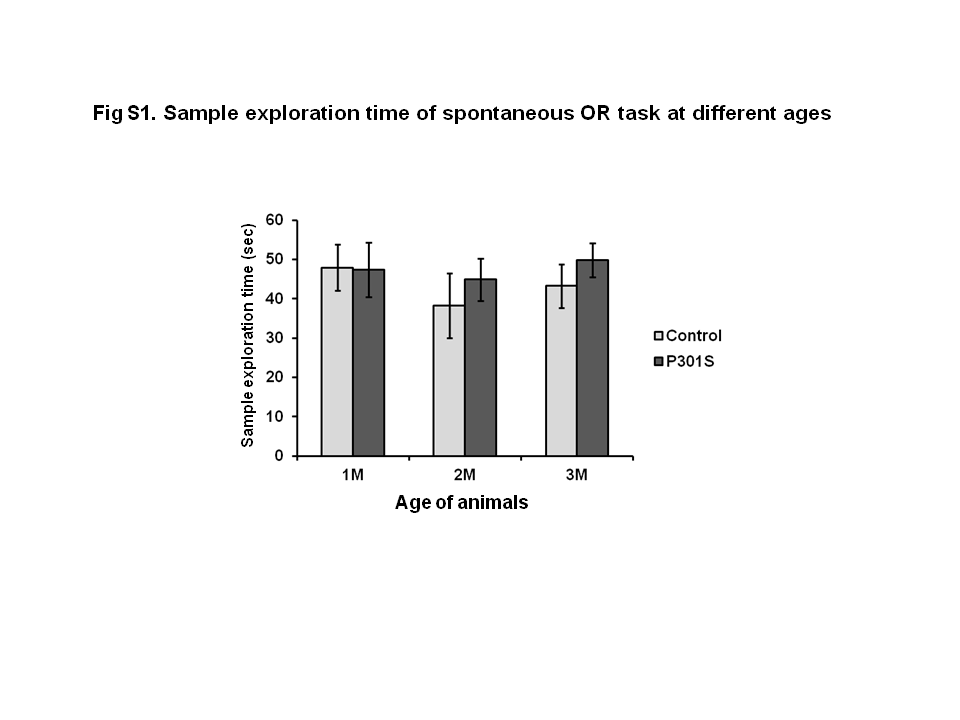

Supplement: Fig. S1 — Sample exploration time for Tg P301S mice at different ages. Sample exploration times were measured in P301S mice and age-matched control mice at 1 M, 2 M and 3 M olds. No difference between P301S mice and control mice was found. Data are presented as mean ± SEM. 1 M: Control n = 7, P301S n = 9; 2 M: Control n = 5, P301S n = 7; 3 M: Control n = 6, P301S n = 10. [file mmc1.zip › XNR11885-mmc1.TIF]

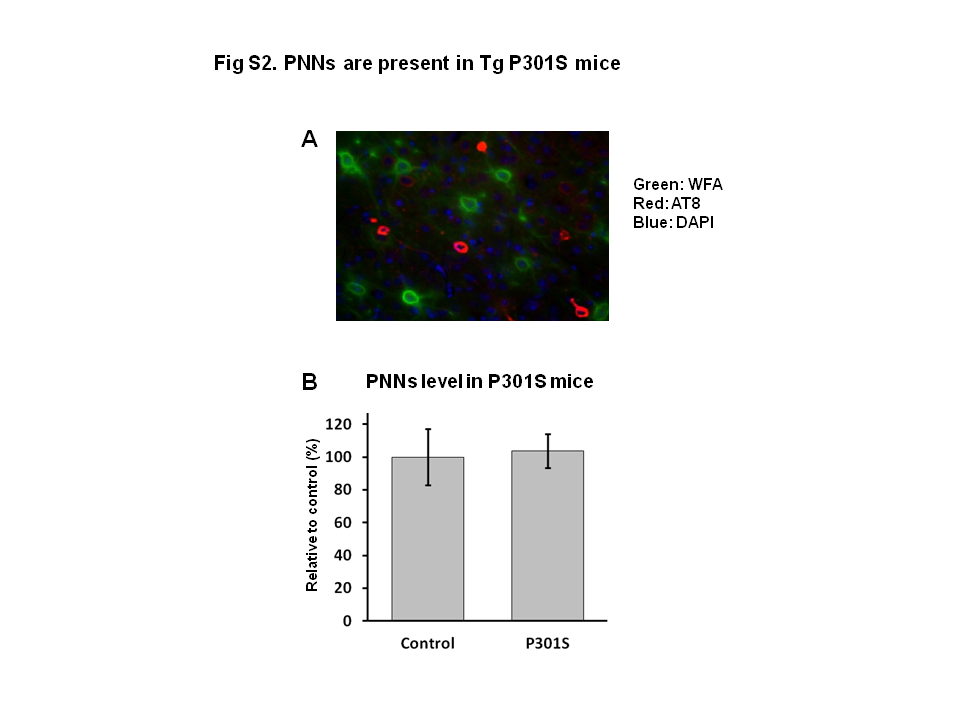

Supplement: Fig. S2 — PNNs are present in Tg P301S mice. A. Brain slices of P301S mice were stained with biotin-WFA and AT8 antibodies. WFA-positive neurons in the PRh were visible in P301S mice. Green: WFA-positive, red: AT8-positive, blue: DAPI B. Stereological analysis of WFA-positive neurons in the PRh of control and P301S mice at 3 month old. Data are presented as mean ± SEM. Control n = 4 P301S n = 6. [file mmc2.zip › XNR11885-mmc2.TIF]

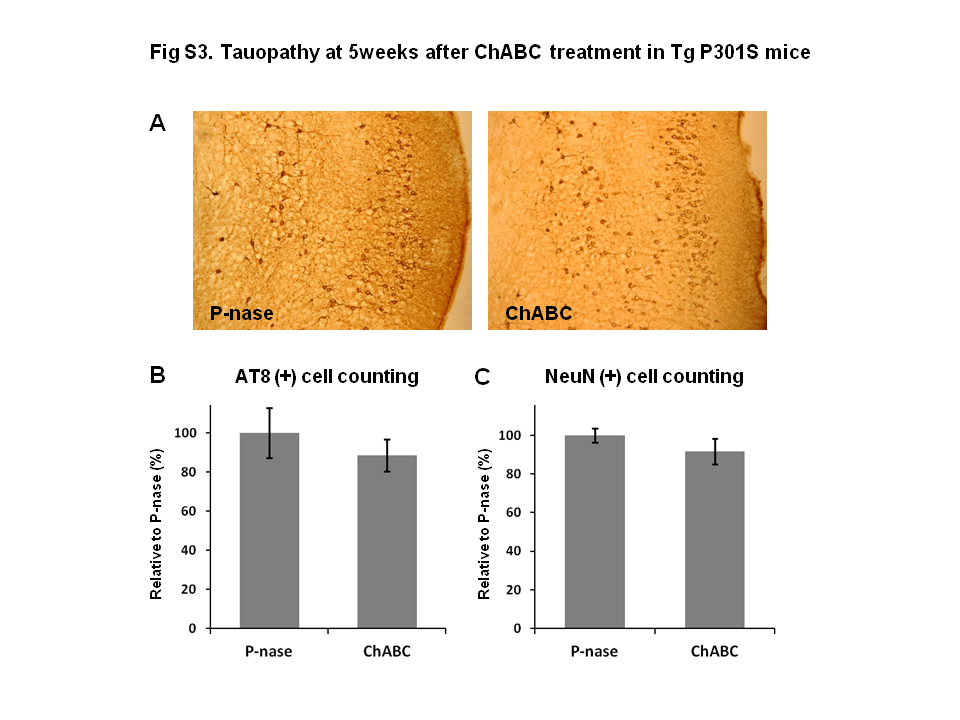

Supplement: Fig. S3 — Tauopathy after 5 weeks of ChABC treatment in Tg P301S mice. A. Hyperphosphorylated tau was visualized by AT8 antibody immunostaining in the PRh of P-nase and ChABC injected mice at 5 weeks post-injection. AT8 (B) or NeuN-positive cells (C) were stereologically quantified in the PRh cortex of P301S mice (P-nase n = 6, ChABC n = 6). No significant influence of ChABC treatment on AT8 staining or neuronal cell number was present in the Tg P301S mice. Data are presented as mean ± SEM. [file mmc3.zip › XNR11885-mmc3.TIF]
